# Sex differences in electrophysiological properties and voltage-gated ion channel expression in the paraventricular thalamic nucleus following repeated stress

**DOI:** 10.1186/s13293-022-00460-0

**Published:** 2022-09-27

**Authors:** Brian F. Corbett, Kimberly Urban, Sandra Luz, Jason Yan, Jay Arner, Seema Bhatnagar

**Affiliations:** 1 Department of Biology, Rutgers, The State University of New Jersey, Camden, NJ USA; 2 Center for Stress Neurobiology, Department of Anesthesiology and Critical Care, Children’s Hospital of Philadelphia, Philadelphia, PA USA; 3 Perelman School of Medicine, University of Pennsylvania, Philadelphia, PA USA

## Abstract

**Background:**

Habituation to repeated stress refers to a progressive reduction in the stress response following multiple exposures to the same, predictable stressor. We previously demonstrated that the posterior division of the paraventricular thalamic nucleus (pPVT) nucleus regulates habituation to 5 days of repeated restraint stress in male rats. Compared to males, female rats display impaired habituation to 5 days of restraint. To better understand how activity of pPVT neurons is differentially impacted in stressed males and females, we examined the electrophysiological properties of pPVT neurons under baseline conditions or following restraint.

**Methods:**

Adult male and female rats were exposed to no stress (handling only), a single period of 30 min restraint or 5 daily exposures to 30 min restraint. 24 h later, pPVT tissue was prepared for recordings.

**Results:**

We report here that spontaneous excitatory post-synaptic current (sEPSC) amplitude was increased in males, but not females, following restraint. Furthermore, resting membrane potential of pPVT neurons was more depolarized in males. This may be partially due to reduced potassium leakage in restrained males as input resistance was increased in male, but not female, rats 24 h following 1 or 5 days of 30-min restraint. Reduced potassium efflux during action potential firing also occurred in males following a single restraint as action potential half-width was increased following a single restraint. Restraint had limited effects on electrophysiological properties in females, although the mRNA for 10 voltage-gated ion channel subunits was altered in the pPVT of female rats.

**Conclusions:**

The results suggest that restraint-induced changes in pPVT activation promote habituation in males. These findings are the first to describe a sexual dimorphism in stress-induced electrophysiological properties and voltage-gated ion channel expression in the pPVT. These results may explain, at least in part, why habituation to 5 days of restraint is disrupted in female rats.

## Highlights

- Male, but not female, pPVT neurons display increases in EPSC amplitude and decay time 24 h following one and five restraints.
- Input resistance is increased 24 h following one and five restraints in male, but not female, pPVT neurons.
- Afterhyperpolarization potential is greater in pPVT neurons of females compared to males, regardless of restraint.
- Restraint alters the expression of 10 voltage-gated ion channel transcripts in the pPVT of females, but only 3 in males.

## Introduction

Habituation to repeated stress refers to a progressive decrease in the stress response following multiple exposures to the same, mild-to-moderately intense homotypic stressor [1–3]. Animals that habituate display reductions in plasma concentrations of adrenocorticotropic hormone (ACTH) and corticosterone as well as stress-related behaviors [1–6]. Habituation is phylogenetically conserved, occurring in animals ranging from rodents [7] to humans [6]. Habituation to stress is considered adaptive, because it allows humans and animals to filter out irrelevant stimuli and focus selectively on important stimuli [8]. Impaired habituation is a hallmark of post-traumatic stress disorder (PTSD) as it contributes to the hyperarousal and re-experiencing symptom clusters [9–11]. Habituation is also a predictor of PTSD treatment efficacy as habituation to in vivo and imaginal exposure of the trauma is associated with the success of prolonged exposure therapy [10, 12, 13]. Effective habituation reduces bioavailable ACTH and corticosterone, thus limiting prolonged exposure to stress-related hormones and other responses that adversely impact physiological functions. Therefore, habituation is a fundamental aspect of the stress response that plays an important role in stress-related disorders. Compared to men, women are approximately twice as likely to be diagnosed with stress-related mood disorders, such as PTSD, depression, and anxiety in Western countries [14–16]. Understanding sex differences in the mechanisms underlying stress habituation may provide insight into the higher prevalence of these stress-related disorders in women.

We previously showed that habituation is dependent on the paraventricular thalamic nucleus (PVT). Chemogenetic inhibition of the posterior division of the PVT (pPVT) or excitatory lesions impair behavioral and neuroendocrine habituation in male rats [7, 17]. The PVT is an extensive midline thalamic nucleus [18–23] that receives afferents from the nucleus tractus solitarius, parabrachial nuclei, locus coeruleus, raphe nuclei, prefrontal cortex, amygdala, and the suprachiasmatic nuclei [20, 22, 24–28]. Projections differ throughout the rostro-caudal axis of the PVT. Anterior (a)PVT projections are widespread [20, 29–31], whereas pPVT projections are more limited and primarily target limbic structures. Efferent projections from the pPVT include the central, basomedial, and basolateral amygdala, nucleus accumbens, anterior olfactory nucleus, bed nucleus of the stria terminalis (BNST), peri-posterior paraventricular nucleus of the hypothalamus (peri-PVN), but not the PVN, and the infralimbic (IL) and prelimbic (PL) divisions of the medial prefrontal cortex (mPFC) [20–22, 24, 26, 27, 32]. In addition to habituation, our previous work has shown that the pPVT mediates facilitated hypothalamic pituitary adrenal (HPA) responses to repeated stress, mediates anxiety-related behaviors and that these responses are specifically mediated by the posterior, but not the anterior, division of the PVT.

Male and female rats display fundamental differences in regulation of the HPA axis. Compared to males, female rats display higher concentrations of corticosterone under baseline conditions and in response to stress [33]. These differences are driven by estradiol, at least in part, as estradiol potentiates adrenocorticotropic hormone (ACTH)-mediated increases in plasma corticosterone concentrations in ovarectomized females [34, 35] and HPA responses to stress are higher during the proestrus phase of the estrous cycle when estradiol concentrations are elevated [36]. Indeed, plasma corticosterone concentrations correlate with estradiol production throughout the estrous cycle [37]. Conversely, androgens inhibit HPA axis activation in gonadectomized males by reducing stress-induced activation of the paraventricular nucleus of the hypothalamus (PVN) and subsequent increases in corticotropin releasing factor in the PVN. Androgen-mediated inhibition of the PVN during stress results in reduced plasma ACTH concentrations and plasma corticosterone concentrations [38].

We previously reported that female rats display impaired habituation of plasma ACTH and corticosterone and behavioral habituation compared to male rats during a 5-day restraint paradigm [33]. To better understand the substrates through which habituation may be regulated differently in male compared to female rats, we examined the electrophysiological properties of the pPVT in males and females. Sex differences in these properties have not been previously examined. Here we examined electrophysiological properties of pPVT neurons using whole cell patch clamp recordings in male and female rats that were either not stressed, restrained once, or restrained for 5 consecutive days. We also examined the mRNA of ion channels in the pPVT that are relevant to some of these electrophysiological properties. Broadly, the results suggest differences in pPVT cells under both baseline and stressed conditions and in ion channel expression that may help to explain why males habituate to 5 days of restraint, whereas female rats do not. Understanding sex differences in habituation, a fundamental aspect of the stress response, may provide insight into the higher rates of stress-related mood disorders in women compared to men.

## Methods

### Animals

Male and female Sprague–Dawley rats at postnatal days 38–45 were ordered from Charles River Laboratories (Wilmington, MA) and allowed to acclimate for 4 days. All rats within each experiment were purchased at the same time and singly housed immediately following delivery. An animal facility employee randomly positioned each rat on a cage rack. A lab member that was not otherwise involved in these experiments randomly assigned a number to each rat, so that they could be separated into one of three groups: non-restrained control, 1-day restraint, or 5-day restraint. Rats in all groups were singly housed throughout the duration of the 5-day restraint paradigm in polycarbonate cages with standard bedding and with food and water available ad libitum. Animals were acclimated to a 12-h light–dark cycle with lights on at 06:15 and lights off at 18:15 in a temperature-controlled vivarium for at least 4 days prior to administration of any restraint stress protocols. All experiments took place during the inactive phase between 1000 and 1400 h. Experiments were performed in compliance with all relevant ethical regulations for animal testing and research. Experiment protocols followed the NIH Guide for the Care and Use of Laboratory Animals and were approved by the Children’s Hospital of Philadelphia Research Institute’s Animal Care and Use Committee.

### Restraint

Rats were randomly separated into one of three groups: control, single-restraint, and 5-day restraint. Controls rats were singly housed until recordings. Restrained rats were placed in a clear, plexiglass tube for 30 min each day. Single restraint rats underwent a single 30-min restraint stress. Repeated restraint rats underwent 5 consecutive, daily 30-min restraints. Restrained rats were singly housed in their home cages as control rats were. All rats were sacrificed 24 h following their final restraint. All restraints were performed at the same time each day (10:00 am EST) to avoid confounding variability in circadian rhythms.

### pPVT slice preparation

Twenty-four hours following the final restraint session, rats were anesthetized using 5% inhaled Isoflurane (Baxter Healthcare) until they no longer responded to a toe pinch. Animals were then rapidly perfused intracardially with ice-cold artificial cerebrospinal fluid (aCSF) containing 87 mM NaCl, 75 mM Sucrose, 25 mM NaHCO_3_, 25 mm glucose, 2.5 mM KCl, 1.25 mM NaH_2_PO_4_, 0.5 mM CaCl_2_, 7 mM MgSO_4_ and ventilated with 95% O_2_/5% CO_2_. The brain was rapidly removed, the frontal lobe and cerebellum sectioned off, and the remaining section placed on a Leica VT1000S vibratome (Leica Microsystems). Once placed on the sectioning stage, the brain was further sectioned, with the cortex trimmed above and on both sides. The optic nerves were sectioned off the ventral side. The remaining block of brain containing paraventricular nucleus of the thalamus was sliced at 300 µm, with a 27° blade angle. Slices were collected once the base of the third ventricle was visible, with a distinct “v” shape. Slices were then incubated at 37.5 °C for 1 h, ventilated with 95% O_2_/5% CO_2_, and then stored at room temperature while bubbling for the remainder of recordings.

### Electrophysiological recordings

**Fig. 1:**
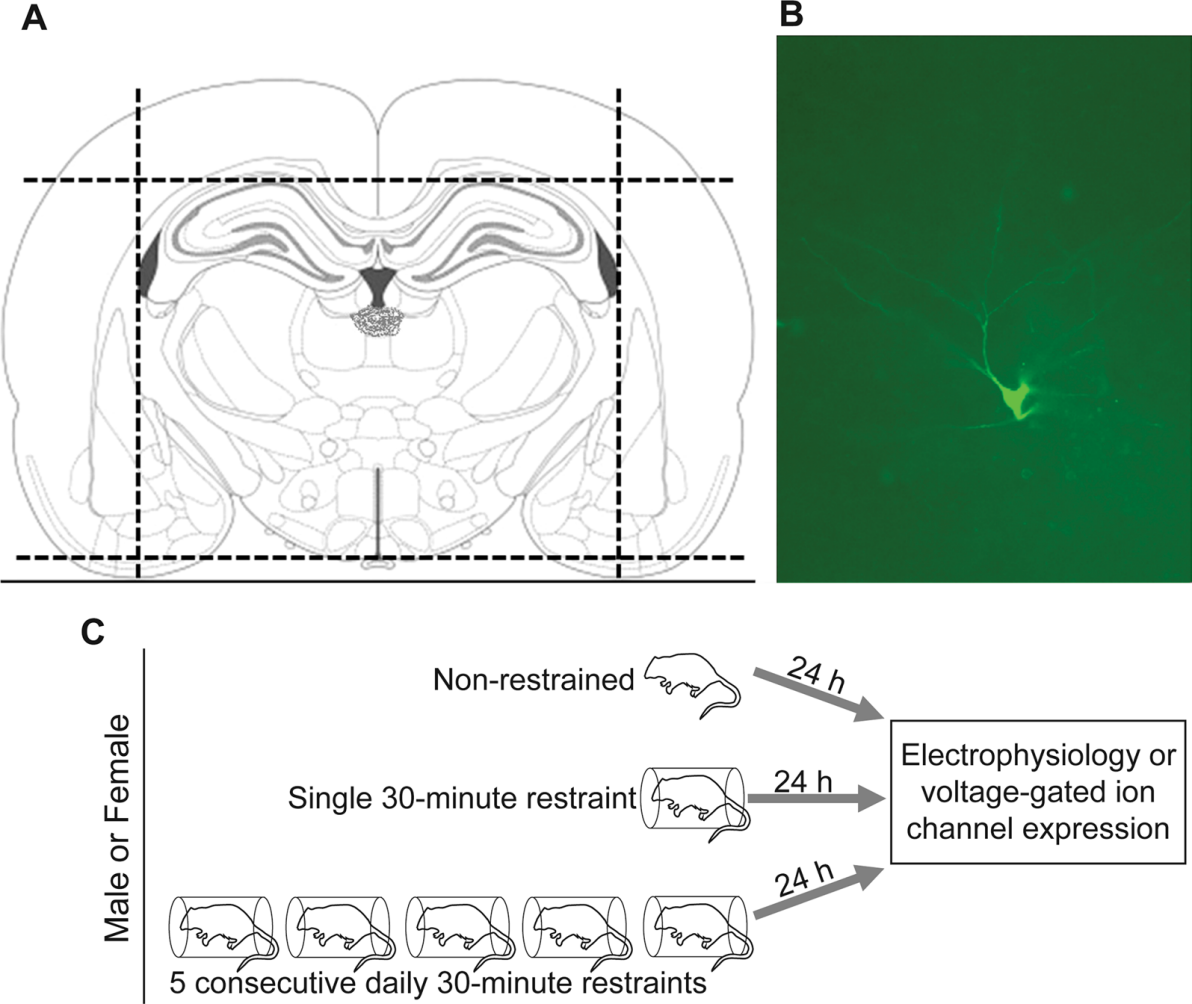
Location of electrophysiological recordings in the pPVT. **A** Schematic showing approximate locations of electrophysiological recordings in the pPVT. **B** Example of biocytin-labeled pPVT neuron used to confirm recording within the pPVT. **C** Male and female rats were either non-restrained controls, restrained once for 30 min, or restrained 30 min each day for 5 consecutive days. Rats were euthanized 24 h following the end of their final restraint and tissue was collected for electrophysiology or quantification of voltage-gated ion channel mRNA

Slices containing posterior paraventricular nucleus of the thalamus (pPVT) were bathed in heated (36–37 °C) Ringer’s aCSF containing 125 mM NaCl, 25 mM NaHCO_3_, 25 mM glucose, 2.5 mM KCl, 1.25 mM NaH_2_PO_4_, 2 mM CaCl_2_, 1 mM MgCl_2_, and ventilated with 95%O_2_/5%CO_2_. Tissue was visualized using differential interference contrast on a Leica Axioskop using the Dage-MTI camera system. The pPVT was located by finding the base of the third ventricle, and searching the butterfly-shaped region directly below (Fig. 1). Glass electrodes were pulled using the Sutter P-97 and filled with an ice-cold potassium gluconate intracellular solution (120 mM K-gluconate, 6 mM KCl, 0.3 mM GTP, 0.2 mM EGTA, 10 mM HEPES, and 4 mM ATP-Mg) containing 0.2% biocytin to label recorded neurons. Whole-cell patching was performed and only neurons with a gigaohm seal were selected for recording and analysis. The Clampex 10.0 software suite (Molecular Devices) was used to write and record programs, the Multiclamp 700B Commander was used to maintain seals and apply current or voltage. For excitability studies, cells were held in current clamp. A step current protocol of 15 steps in 50pA increments, from − 300 to 300 pA, was applied, with each sweep lasting 30 s. Passive membrane properties (input resistance and resting membrane potential) were recorded and analyzed, as well as number of action potentials, threshold, peak amplitude, half-width of the action potentials, and amplitude of afterhyperpolarization, using Clampfit 10.0 (Axon Laboratories). Following the step current protocol, cells were switched to voltage clamp and held at − 60 mV, and spontaneous excitatory postsynaptic currents (sEPSCs) were recorded for 5 min to assess synaptic transmission. Frequency and amplitude of sEPSCs were recorded and analyzed using Clampfit 10.0 (Axon Laboratories).

### Ion channel microarrays

Two cohorts of 24 rats, one male and one female were randomly separated into one of three groups: control, single-restraint, or repeated restraint, with 8 animals in each group. Single restraint rats underwent a single 30-min restraint. Repeated restraint rats underwent 5 consecutive days of 30-min restraint stress. Twenty-four hours after the final restraint, animals were sacrificed, brains were rapidly removed and flash-frozen. Frozen brains were fixed to a cryostat stage and the pPVT was microdissected. RNA was isolated using the RNeasy Plus MicroKit (74034, Qiagen) and carried out per the manufacturer's instructions. Approximately 500 ng of RNA was collected per pPVT sample. Reverse transcription of isolated pPVT RNA was performed using the RT2 First Strand Kit (330404, Qiagen). A Rat Neuronal Ion Channel RT2 Profiler PCR array (PARN-036ZA, Qiagen) containing SYBR Green RT PCR assays for 84 genes of interest, 5 housekeeping genes (Beta-actin, Ribosomal protein large P1, hypoxanthine phosphoribosyltransferase 1, lactate dehydrogenase A, and glyceraldehyde 3-phosphate dehydrogenase) and 3 synthetic control genes (reverse transcription control, positive PCR control and rat genomic DNA contamination control) was used to run quantitative PCR on an ABI 7500 PCR system. Samples were run according to manufacturer’s instructions and analyzed as previously described [39]. The comparative Ct method was used to assess mRNA expression differences for genes of interest. This generated a fold change value with the expression of 1-day restraint and 5-day restraint groups relative to non-restrained controls. These fold change values were then normalized to the mean expression levels of the five housekeeping genes for each rat, so that relative levels of each transcript could be estimated.

### Data analysis

All raw electrophysiology data were analyzed using Clampfit 10.0. For analysis of electrophysiology data, two-way ANOVAs were run to compare male and female non-restrained control, 1-day restraint, and 5-day restraint rats. Sex, stress, and the interaction of sex and stress were used as variables. Post hoc comparisons were performed using Tukey’s multiple comparisons test unless otherwise noted. For analysis of mRNA expression, males and females were analyzed separately as they were restrained in different cohorts. To identify potentially significant effects of restraint on mRNA expression, Student’s *t* test was used to identify restraint group differences within each sex at *p* < 0.05 for each transcript. These potentially significant differences were then subjected to an ordinary one-way ANOVA with p-corrected post-hoc testing. An ordinary one-way ANOVA was used to detect differences among non-restrained, 1-day restraint, and 5-day restraint groups within each sex. Dunnett’s multiple comparisons test was used to determine whether 1-day restraint and/or 5-day restraint groups were different from non-restrained controls. Alpha was adjusted to 0.01 to account for multiple comparisons. Any data that were not within three standard deviations from the mean were discarded.

## Results

### Effects of sex and restraint on excitatory postsynaptic currents in the pPVT

**Fig. 2:**
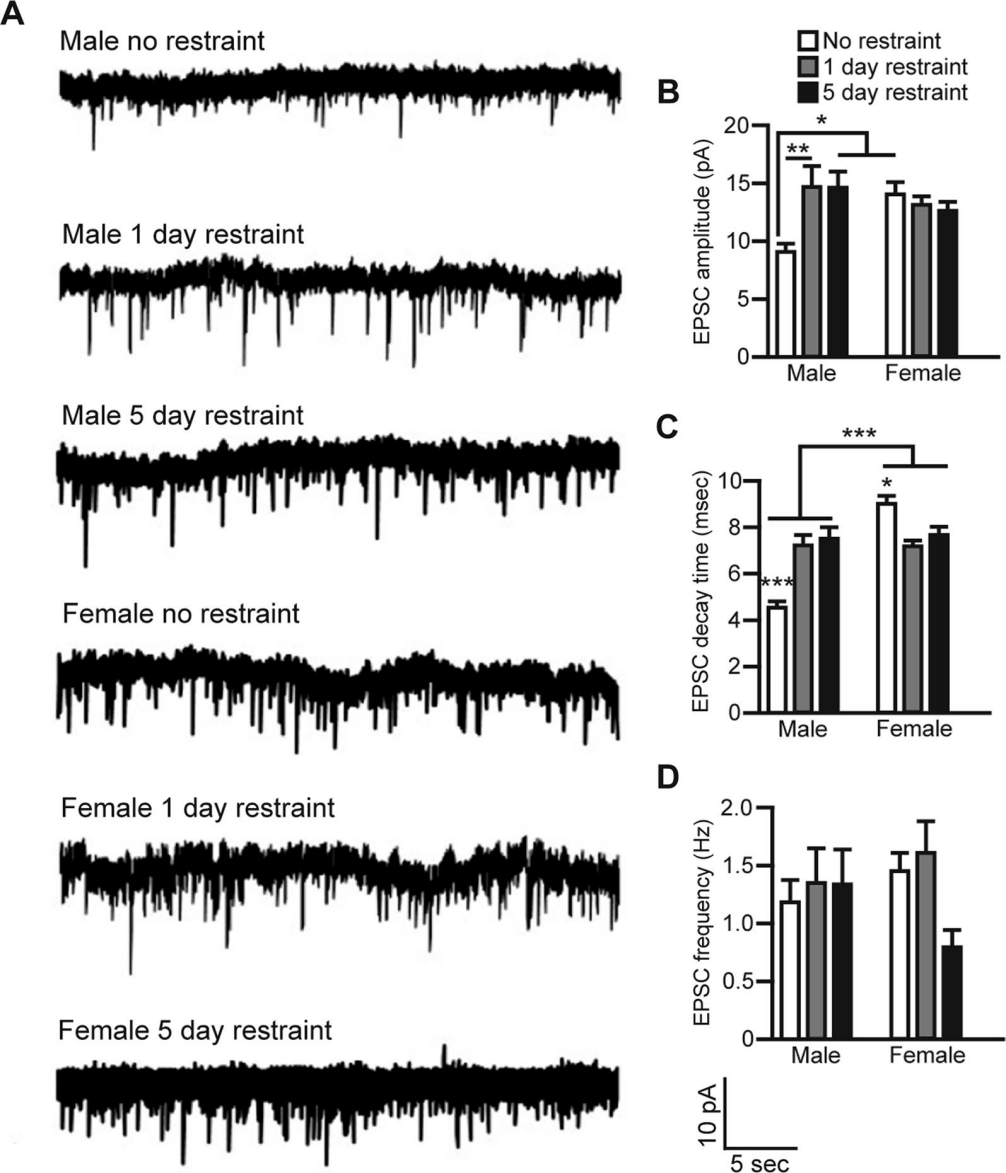
Males and females display differences in sEPSCs in the pPVT at baseline and following restraint. **A** Representative traces of sEPSCs in male and female pPVT neurons 24 h following no restraint, 1 day of restraint, or 5 days of restraint. **B** Amplitude of sEPSCs in the pPVT was reduced in non-restrained males compared non-restrained females. Restraint increased sEPSC amplitude in males, but not females. **C** sEPSC decay time was different between males and females as sex and interaction effects were observed. Post-hoc analysis revealed that sEPSC decay time in non-restrained males was significantly lower than all other groups. **D** No effects of sex or stress were observed on sEPSC frequency. Bars indicate mean ± SEM. For **B**, horizontal lines indicate groups significantly different from non-restrained males following post-hoc analysis. For **C**, horizontal lines indicate sex effect, asterisks indicate differences from other restraint groups within each sex. *p < 0.05, **p < 0.01, ***p < 0.0001. For **B**–**D**, male no restraint *n* = 21, male 1-day restraint *n* = 17, male 5-day restraint *n* = 16, female no restraint *n* = 24, female 1-day restraint n = 17, female 5-day restraint *n* = 17

A trend for a restraint effect on EPSC amplitude was observed (*F*(2,106) = 2.8, *p* = 0.0653), but no sex effect was observed. A significant interaction for sex and restraint was observed for EPSC amplitude in pPVT neurons (*F*(2,106) = 6.471, *p* = 0.0022) (Fig. 2A, B). Post-hoc analysis revealed that this effect was primarily driven by the non-restrained male group. Compared to non-restrained males, an increase in EPSC amplitude was observed in males restrained for 1 (*p* = 0.008) or 5 (*p* = 0.0101) days. Non-restrained females also displayed greater EPSC amplitudes compared to non-restrained males (*p* = 0.0118). Restraint stress had no effect on EPSC amplitude in the pPVT in female rats. Though both acutely and repeatedly restrained females exhibited higher EPSC amplitudes than the control males, these were not significant. Together, these findings demonstrate that EPSC amplitude is higher in females than males under non-stress conditions, both acute and repeated restraint significantly increase EPSC amplitude in males, so that they are similar to the EPSC amplitudes of females, but neither acute nor repeated restraint stress impacts EPSC amplitude of pPVT neurons in female rats.

A main effect of restraint effect was observed for EPSC decay time (*F*(2,106) = 3.359, *p* = 0.0385). An overall sex effect was also observed (*F*(1,106) = 33.1, *p* < 0.0001) with females displaying longer EPSC decay times in pPVT neurons compared to males. A significant interaction effect for sex and restraint was observed for EPSC decay time in pPVT neurons of male and female rats (*F*(2,106) = 35.01, *p* < 0.0001) (Fig. 2C). Post-hoc analysis revealed that in males, 1 and 5 days of restraint increased EPSC decay time compared to non-restrained controls (*p* < 0.0001 for both comparisons). Non-restrained females displayed longer EPSC decay times compared to non-restrained males (*p* < 0.0001). Compared to non-restrained female controls, 1 (*p* = 0.0008) and 5 (*p* = 0.0275) days of restraint reduced EPSC decay time in female rats. No effects of stress, restraint, or their interaction were observed for EPSC frequency (Fig. 2D). Together, our findings indicate that pPVT neuron EPSC decay times are longer in non-restrained females compared to non-restrained males. Furthermore, pPVT neuron EPSC decay time is increased by restraint in males, but decreased by restraint in females. Thus, in male and female rats, pPVT neuron EPSC decay times are different at baseline and oppositely affected by restraint stress. Together, these findings demonstrate that EPSC amplitude in pPVT neurons is higher in females compared to males at baseline. One or 5 days of restraint increased EPSC amplitude in male rats, but not in female rats. In addition, restraint increased pPVT neuron EPSC decay times in males, but restraint decreased EPSC decay times in females.

### Effects of sex and restraint on passive membrane properties of pPVT neurons

**Fig. 3:**
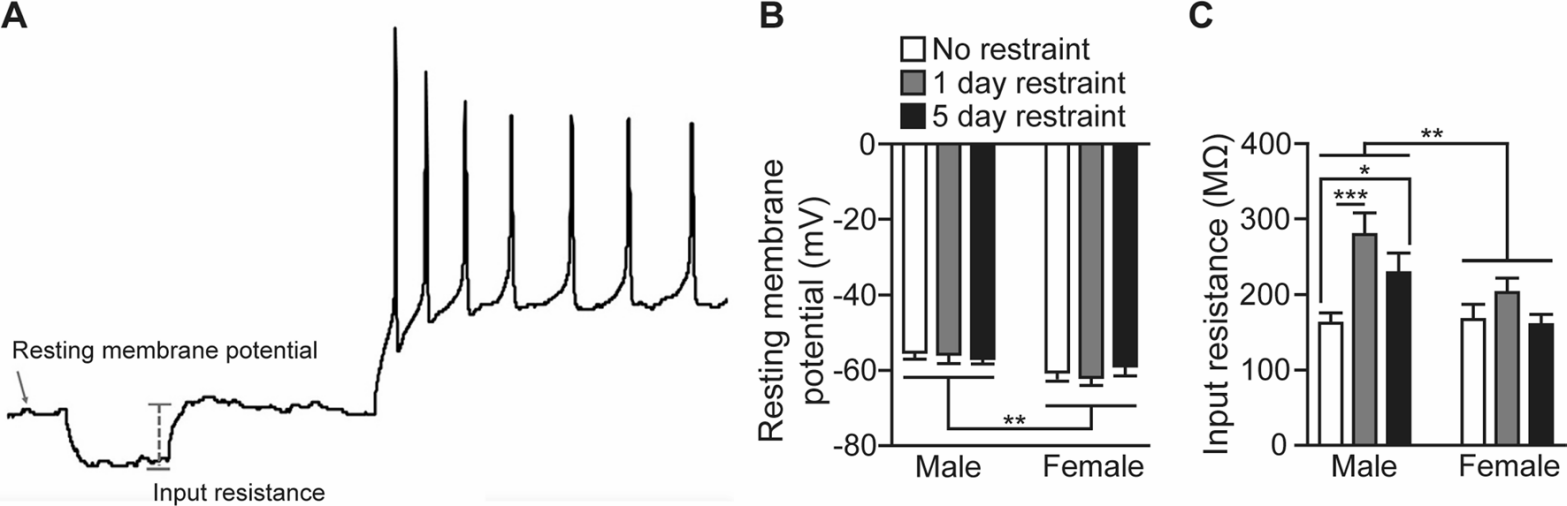
Males and females display differences in passive membrane properties in the pPVT at baseline and following restraint. **A** Schematic of resting membrane potential, a change in resting membrane potential during a hyperpolarizing current used to calculate input resistance, and membrane potential during action potential firing. **B** Females display a more hyperpolarized resting membrane potential compared to males regardless of restraint. **C** input resistance is increased by 1 or 5 restraints in males, but not females, causing a sex effect with males displaying greater input resistance. Bars indicate mean ± SEM. Horizontal bars over each sex represent sex differences. For **C**, horizontal bars within the male group represent post-hoc differences. *p < 0.05, **p < 0.01, ***p < 0.001. For **B** and **C**, male no restraint *n* = 38, male 1-day restraint *n* = 16, male 5-day restraint *n* = 21, female no restraint *n* = 24, female 1-day restraint *n* = 17, female 5-day restraint *n* = 17

We observed a significant main effect of sex for pPVT neuron resting membrane potential but no other effects. pPVT neurons were hyperpolarized in females compared to males (*F*(2,127) = 8.166, *p* = 0.005) (Fig. 3B). For input resistance, a significant sex effect was observed as males displayed higher input resistance of pPVT neurons across all restraint groups compared to females (*F*(1,26) = 8.74, *p* = 0.0037). A restraint effect was also observed (*F*(2,126) = 8.171, *p* = 0.0005). A significant interaction effect for sex and restraint was observed (*F*(2,126) = 3.324, *p* = 0.0392) (Fig. 3C). Male rats that were restrained for 1 (*p* = 0.0002) or 5 (*p* = 0.0472) day displayed increased input resistance of pPVT neurons compared to non-restrained males. No significant post-hoc differences in input resistance among restraint groups were observed within the female group. Together, these findings demonstrate that pPVT neuron resting membrane potential is hyperpolarized in female rats. In addition, 1 or 5 days of restraint increased input resistance in male, but not female, rats.

### Effects of sex and restraint on active membrane properties of pPVT neurons

**Fig. 4:**
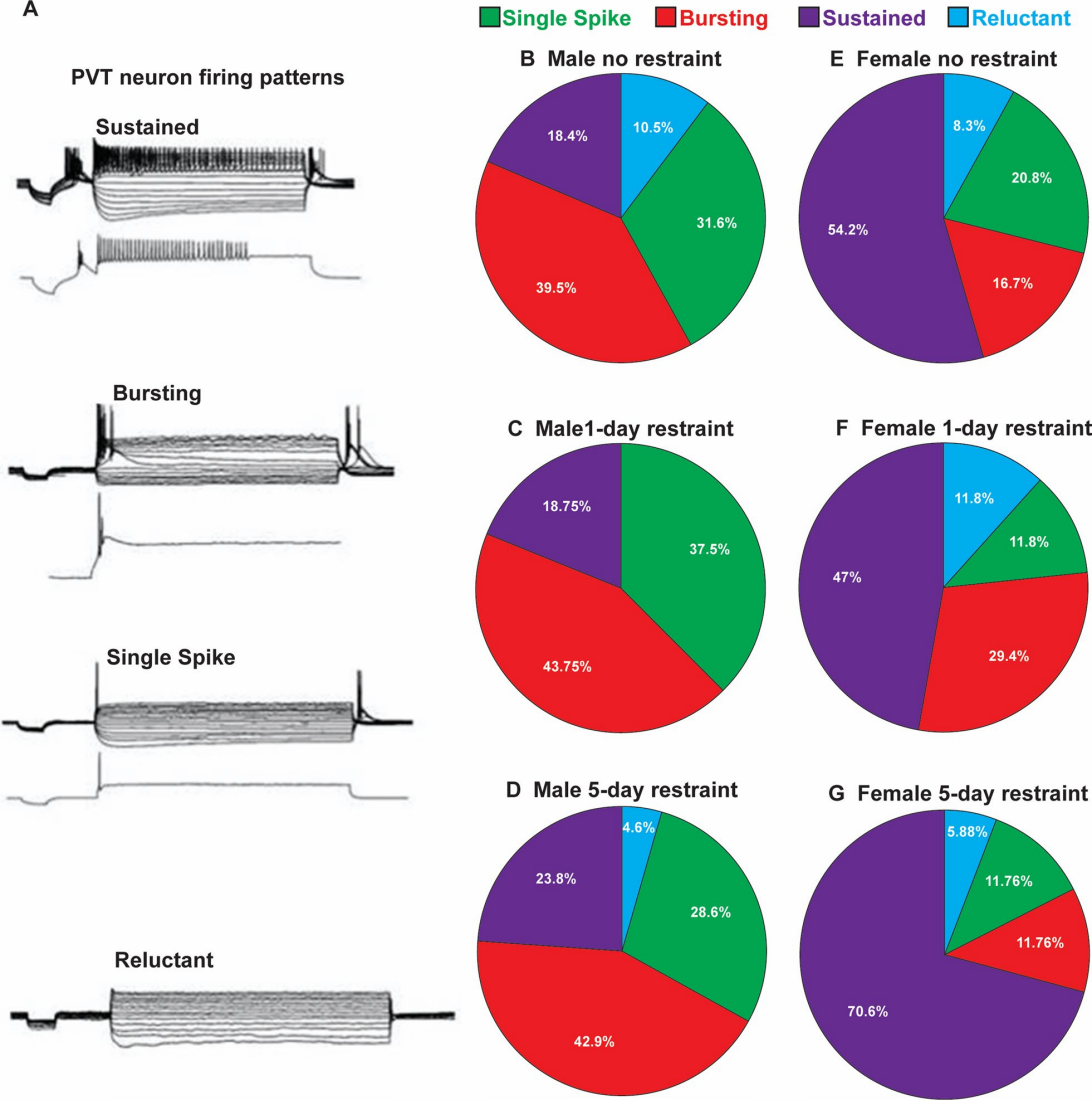
Effects of restraint stress and sex on pPVT neuron firing patterns. **A** Traces of action potential firing patterns in PVT neurons that are characterized as sustained, burst, single, or reluctant firing. The percentage of PVT neurons characterized as sustained, burst, single, or reluctant firing in **B** non-restrained males, **C** 1-day restraint males, **D** 5-day restraint males, **E** non-restrained females, **F** 1-day restraint females, and **G** 5-day restraint females. Male no restraint *n* = 38, male 1-day restraint *n* = 16, male 5-day restraint *n* = 21, female no restraint *n* = 24, female 1-day restraint *n* = 17, female 5-day restraint *n* = 17

We characterized the firing patterns of pPVT neurons as sustained, bursting, single, or reluctant (Fig. 4A) in male and female rats that were either non-restrained controls, restrained for 1 day, or restrained for 5 consecutive days. An overall difference in firing pattern among all six groups was detected (Fig. 4B–G, *χ*^2^(15) = 26.57, *p* = 0.033). Within each restraint group, males displayed a higher percentage of pPVT neurons that were either single spiking (non-restrained: male = 31.6%, female = 20.8%; 1-day restraint: male = 37.5%, female = 11.8%; 5-day restraint: male = 28.6%, female = 11.76%) or bursting (non-restraint: male = 39.5%, female = 16.7%; 1-day restraint: male = 43.75%, female = 29.4%; 5-day restraint: male = 42.9%, female = 11.76%) compared to females (Fig. 4). Compared to male rats, females displayed a higher percentage of pPVT neurons that displayed sustained firing within each restraint group (non-restrained: males = 18.4%, females = 54.2%; 1-day restraint: males = 18.75%, females = 47%; 5-day restraint: males = 23.8%, females = 70.6%). Only a small percentage of pPVT neurons in each group were reluctant to fire (0–11.8%). Percentages of pPVT neuron subtype were generally unaffected by restraint in male rats. In females, the percentage of sustained firing PVT neurons was higher in 5-day restrained rats compared to non-restrained controls and rats restrained for 1 day (70.6% compared to 54.2% and 47%, respectively). Restraint reduced the percentage of single spiking pPVT neurons in females as non-restrained, 1-day restraint, and 5-day restraint females exhibited 20.8%, 11.8%, and 11.76% of pPVT neurons classified as single spiking, respectively. Females restrained for 1 day displayed a higher percentage of bursting pPVT neurons compared to non-restrained controls and females restrained for 5 days (29.4% compared to 16.7% and 11.76%, respectively). Together, these findings suggest that males display higher percentages of single spike and bursting pPVT neurons compared to females, whereas females display higher percentages of sustained firing pPVT neurons. In addition, restraint has little effect on firing pattern changes in the pPVT neurons of males, whereas restraint alters the firing patterns of PVT neurons in females.

**Fig. 5:**
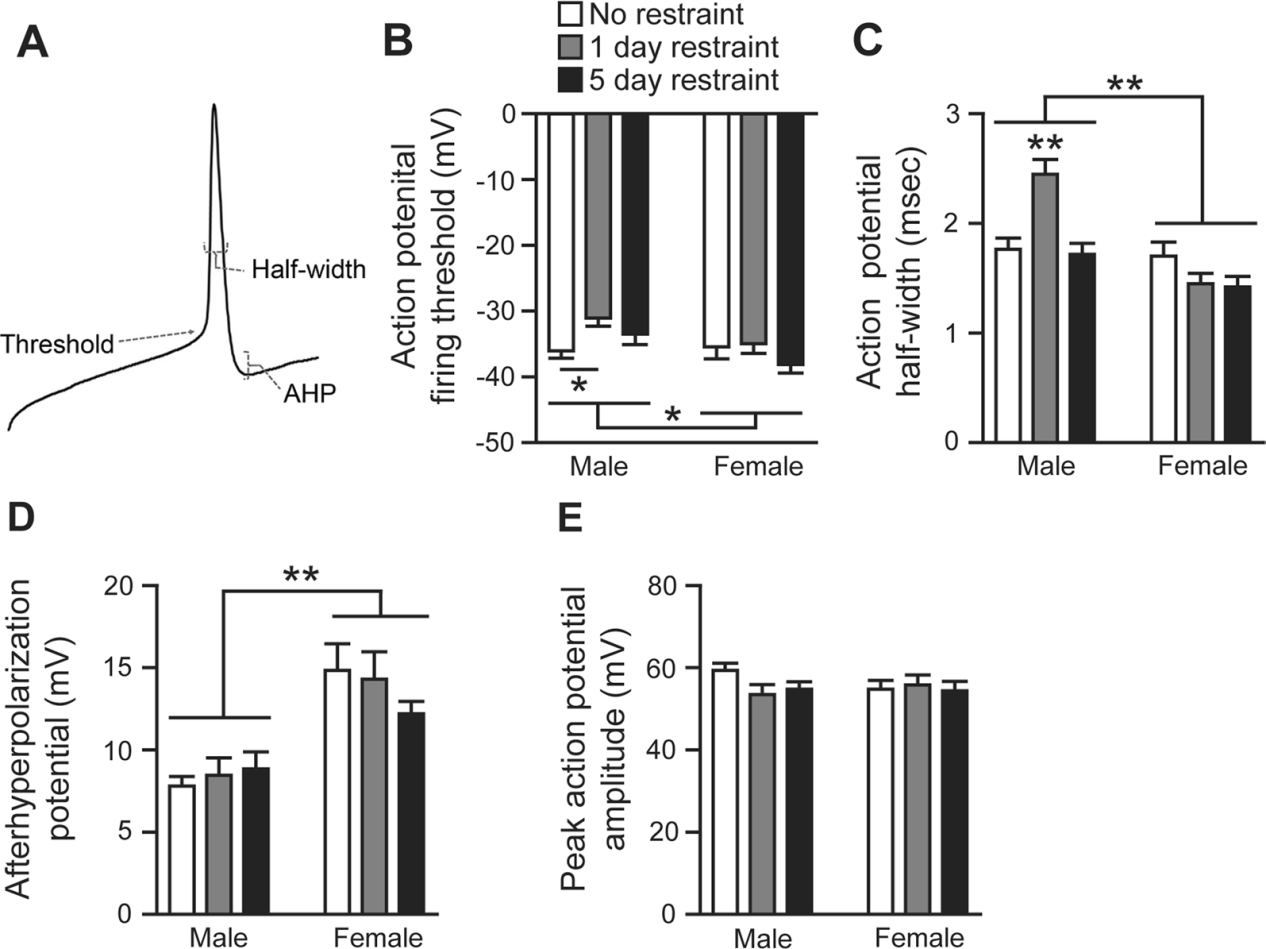
Males and females display differences in active membrane properties in the pPVT at baseline and following a single restraint **A** Schematic showing the aspects of the action potential related to firing threshold, half-width, and afterhyperpolarization potential (AHP). **B** Action potential firing threshold is depolarized 24 h following a single restraint in male pPVT neurons and more hyperpolarized in females compared to males. **C** Action potential half-width is increased in male pPVT neurons 24 h following 1, but not 5, restraints. **D** AHP is increased in female pPVT neurons compared to males regardless of restraint. **E** Peak action potential amplitude is not affected by sex or restraint in the pPVT. Bars indicate mean ± SEM. Horizontal bars over each sex represent sex differences. For **B**, horizontal bar within the male group represents post-hoc difference. For **C**, asterisks above the 1-day restraint male group represent post-hoc differences from all other groups. *p < 0.05, **p < 0.0001. Male no restraint *n* = 34, male 1-day restraint *n* = 16, male 5-day restraint *n* = 20, female no restraint *n* = 22, female 1-day restraint *n* = 15, female 5-day restraint *n* = 16

Main effects for restraint (*F*(2,116) = 3.076, *p* = 0.0499) and sex (*F*(1,116) = 6.074, *p* = 0.0152) and a significant sex × restraint interaction were observed (*F*(2,116) = 3.176, *p* = 0.0454) were observed for action potential firing threshold (Fig. 5A, B) in male or female rats that were either non-restrained controls, restrained for 1 day, or restrained for 5 days (Fig. 5B). Post-hoc analysis revealed the only significant difference within sex or restraint groups was that action potential firing threshold was more depolarized in male rats restrained for 1 day compared to non-restrained male controls (*p* = 0.0350). In general, females displayed a more hyperpolarized action potential firing threshold, although no post-hoc sex differences were observed within any restraint group. For action potential half width, main effects for restraint (*F*(2,117) = 5.529, *p* = 0.0051) and sex (*F*(1,117) = 30.11, *p* < 0.0001) and a significant interaction were observed (*F*(2,117) = 11.26, *p* < 0.0001) (Fig. 5C). Post-hoc analysis revealed that action potential half-width duration was longer in male rats restrained for 1 day compared to all other male and female restraint groups (*p* < 0.0001 for all comparisons). For afterhyperpolarization potentials, a significant sex effect was observed (*F*(1,117) = 35.93, *p* < 0.0001) with females displaying greater afterhyperpolarization potentials compared to males (Fig. 5D). No restraint or interaction effects were observed for afterhyperpolarization potential. No effects of sex, restraint, or their interaction were observed for peak action potential amplitude (Fig. 5E). Together, these findings indicate that a single restraint in male, but not female, rats increases the membrane depolarization required for action potential firing and action potential half-width but this effect in males is not apparent after 5 days of restraint. Furthermore, afterhyperpolarization potential is increased in females compared to their respective male groups regardless of restraint. Therefore, pPVT neurons of males and females display different active membrane properties at baseline and in response to restraint.

### Effects of a single or repeated restraint on voltage-gated ion channel expression in the pPVT of males and females

**Fig. 6:**
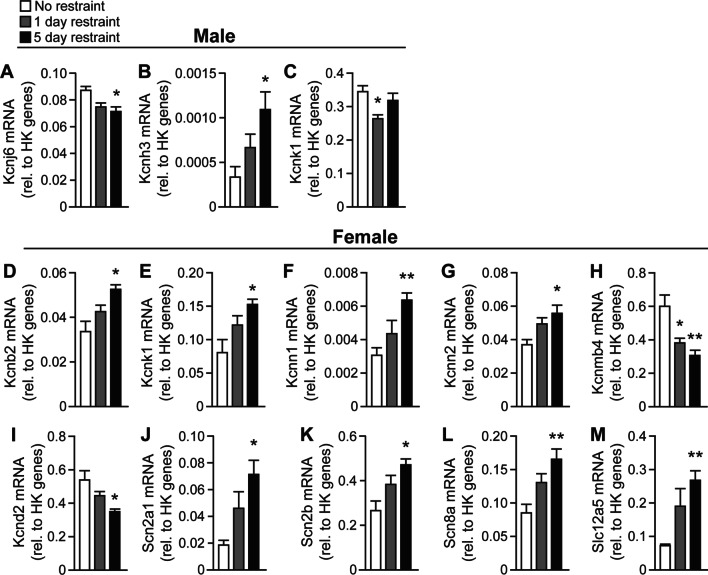
Restraint stress has different effects on the expression of voltage-gated ion channel mRNA transcripts in the PVT of males and females. Only results from males are shown in **A**–**C** and only from females from **D**–**M** to streamline this figure, since results from females were not significant in **A**–**C** and results from males were not significant from **D**–**M**. Results are not significant **A** In male pPVT neurons, Kcnj6 mRNA is reduced in rats restrained for 5 days compared to non-restrained controls. **B** In male pPVT neurons, Kcnh3 mRNA is increased in rats restrained for 5 days compared to non-restrained controls. **C** In male pPVT neurons, Kcnk1 mRNA is reduced following 1, but not 5, restraints compared to non-restrained controls. Females restrained for 5 days display increased mRNA expression of **D** Kcnb2, **E** Kcnk1, **F** Kcnn1, and **G** Kcnn2 in the pPVT compared to non-restrained controls. Females restrained for 5 days display decreased mRNA expression of **H** Kcnmb4 and **I** Kcnd2 in the pPVT compared to non-restrained controls. Females restrained for 5 days display increased mRNA expression of **J** Scn2a1, **K** Scnb2, **L** Scn8a, and **M** Slc12a5 in the pPVT compared to non-restrained controls. Bars indicate mean ± SEM. Asterisks indicate Dunnett’s post-hoc differences compared to non-restrained controls following one-way ANOVA. *p < 0.01, **p < 0.001. For non-restrained males, *n* = 7. For all other groups, *n* = 8

We quantified the mRNA transcripts of voltage-gated ion channels in the pPVT of male (Table 1) and female (Table 2) rats that were either not restrained, or in animals at 24 h after a single restraint or after 5 daily restraints. In male rats, mRNA of the voltage-gated potassium channel Kcnj6, which encodes the G-protein rectifier potassium channel 2, was decreased 24 h following 5 restraints compared to non-restrained males (Fig. 6A, *F*(2,20) = 6.322, *p* = 0.0079; post-hoc *p* = 0.0054). Expression of Kcnh3, which encodes the voltage-gated potassium channel subunit Kv12.2, was increased in male rats 24 h following a 5th restraint compared to non-restrained males (Fig. 6B, *F*(2,20) = 5.055, *p* = 0.0167; post-hoc *p* = 0.0093). Expression of Kcnk1, which encodes a 2-pore domain subfamily K potassium channel, was decreased 24 h following a single restraint, but not 5 restraints, compared to non-restrained males (Fig. 6C, *F*(2,20) = 5.294, *p* = 0.0149; post-hoc *p* = 0.0092).

**Table 1:** Voltage-gated ion channel mRNA expression in male rats following restraint

Males	Mean			SEM			*p* value		
Gene	NR	1D	5D	NR	1D	5D	NR vs. 1D	NR vs. 5D	1D vs. 5D
Accn1	0.0676	0.0658	0.0716	0.0039	0.0035	0.0045	0.7373	0.5103	0.3196
Accn2	0.0781	0.0786	0.0798	0.0029	0.0050	0.0046	0.9282	0.7687	0.8703
Accn3	0.0014	0.0014	0.0013	0.0002	0.0001	0.0001	0.8903	0.6661	0.4797
Best1	0.0038	0.0038	0.0038	0.0002	0.0002	0.0003	0.7875	0.8777	0.9623
Cacna1a	0.0178	0.0156	0.0153	0.0020	0.0021	0.0013	0.4675	0.2942	0.8986
Cacna1b	0.0195	0.0168	0.0163	0.0019	0.0020	0.0011	0.3468	0.1586	0.8366
Cacna1c	0.0523	0.0386	0.0386	0.0050	0.0030	0.0028	0.0311	0.0285	0.9938
Cacna1d	0.0367	0.0347	0.0380	0.0023	0.0027	0.0023	0.5958	0.6913	0.3691
Cacna1g	0.1123	0.0869	0.0797	0.0142	0.0115	0.0085	0.1832	0.0633	0.6223
Cacna1i	0.0671	0.0608	0.0563	0.0056	0.0039	0.0041	0.3697	0.1402	0.4418
Cacnb1	0.0610	0.0476	0.0587	0.0056	0.0061	0.0033	0.1331	0.7243	0.1302
Cacnb2	0.1410	0.1249	0.1196	0.0088	0.0103	0.0070	0.2626	0.0760	0.6807
Cacnb3	1.6532	1.5990	1.7831	0.1878	0.1588	0.1252	0.8278	0.5660	0.3782
Cacng2	0.0822	0.0649	0.0624	0.0081	0.0092	0.0059	0.1891	0.0653	0.8211
Cacng4	0.2373	0.2242	0.2128	0.0143	0.0180	0.0125	0.5876	0.2185	0.6114
Clcn2	0.0554	0.0556	0.0518	0.0030	0.0048	0.0026	0.9761	0.3670	0.4966
Clcn3	0.2162	0.2086	0.2116	0.0125	0.0212	0.0209	0.7723	0.8604	0.9211
Clcn7	0.1178	0.1054	0.1055	0.0040	0.0056	0.0035	0.1044	0.0362	0.9904
Hcn1	0.0159	0.0130	0.0121	0.0014	0.0018	0.0015	0.2377	0.0919	0.6971
Hcn2	0.1661	0.1463	0.1408	0.0115	0.0124	0.0110	0.2692	0.1369	0.7426
Kcna1	0.0180	0.0154	0.0156	0.0012	0.0028	0.0024	0.4306	0.4047	0.9633
Kcna2	0.4879	0.4318	0.4557	0.0358	0.0238	0.0289	0.2041	0.4919	0.5335
Kcna5	0.0179	0.0156	0.0231	0.0018	0.0011	0.0015	0.2701	0.0448	0.0013
Kcna6	0.1564	0.1455	0.1361	0.0093	0.0106	0.0064	0.4573	0.0885	0.4631
Kcnab1	0.0510	0.0465	0.0390	0.0087	0.0110	0.0052	0.7581	0.2422	0.5439
Kcnab2	0.1595	0.1456	0.1324	0.0134	0.0157	0.0091	0.5197	0.1117	0.4795
Kcnab3	0.0270	0.0266	0.0249	0.0021	0.0011	0.0013	0.8638	0.4124	0.3519
Kcnb1	0.1371	0.1303	0.1288	0.0068	0.0051	0.0054	0.4362	0.3586	0.8446
Kcnb2	0.0650	0.0587	0.0584	0.0054	0.0032	0.0039	0.3209	0.3355	0.9611
Kcnc1	0.0774	0.0617	0.0597	0.0096	0.0098	0.0056	0.2756	0.1233	0.8597
Kcnc2	0.1879	0.1375	0.1223	0.0336	0.0285	0.0156	0.2707	0.0877	0.6472
Kcnd2	0.4252	0.4100	0.3910	0.0206	0.0173	0.0120	0.5774	0.1619	0.3822
Kcnd3	0.1127	0.1053	0.1161	0.0071	0.0055	0.0080	0.4157	0.7654	0.2885
Kcnh1	0.0191	0.0199	0.0226	0.0018	0.0018	0.0022	0.7410	0.2390	0.3551
Kcnh2	0.0342	0.0287	0.0320	0.0033	0.0030	0.0036	0.2297	0.6604	0.4897
Kcnh3	0.0003	0.0007	0.0011	0.0001	0.0002	0.0002	0.1108	0.0092	0.1241
Kcnh6	0.0141	0.0147	0.0152	0.0012	0.0017	0.0010	0.7631	0.4673	0.8038
Kcnh7	0.0568	0.0465	0.0475	0.0041	0.0041	0.0044	0.1003	0.1539	0.8661
Kcnj1	0.0005	0.0006	0.0006	0.0000	0.0001	0.0001	0.2627	0.3344	0.8005
Kcnj11	0.0240	0.0208	0.0237	0.0021	0.0013	0.0018	0.1963	0.9101	0.2121
Kcnj12	0.0491	0.0425	0.0559	0.0056	0.0037	0.0069	0.3299	0.4714	0.1106
Kcnj13	0.0403	0.0258	0.0155	0.0171	0.0107	0.0076	0.4751	0.1894	0.4446
Kcnj14	0.0145	0.0167	0.0158	0.0006	0.0014	0.0012	0.1869	0.3671	0.6338
Kcnj15	0.0007	0.0005	0.0015	0.0001	0.0002	0.0008	0.6058	0.3390	0.2512
Kcnj16	0.1199	0.1195	0.1114	0.0104	0.0138	0.0092	0.9822	0.5481	0.6309
Kcnj2	0.0051	0.0054	0.0057	0.0003	0.0003	0.0004	0.3667	0.2099	0.5206
Kcnj3	0.0735	0.0676	0.0603	0.0055	0.0073	0.0044	0.5360	0.0807	0.4098
Kcnj4	0.0032	0.0032	0.0033	0.0006	0.0005	0.0003	0.9778	0.9063	0.9284
Kcnj5	0.0140	0.0161	0.0143	0.0013	0.0021	0.0011	0.4352	0.8695	0.4650
Kcnj6	0.0872	0.0748	0.0713	0.0029	0.0028	0.0038	0.0118	0.0063	0.4895
Kcnj9	0.1456	0.1261	0.1103	0.0172	0.0178	0.0094	0.4481	0.0842	0.4452
Kcnk1	0.3452	0.2637	0.3189	0.0160	0.0152	0.0197	0.0039	0.3280	0.0536
Kcnma1	1.0209	1.0620	1.0787	0.0962	0.1175	0.0893	0.7948	0.6667	0.9114
Kcnmb4	0.1986	0.1801	0.1832	0.0129	0.0153	0.0085	0.3786	0.3243	0.8619
Kcnn1	0.0051	0.0047	0.0044	0.0002	0.0003	0.0003	0.3390	0.1165	0.5635
Kcnn2	0.0795	0.0836	0.0754	0.0055	0.0051	0.0038	0.5887	0.5440	0.2178
Kcnn3	0.1135	0.1048	0.1092	0.0070	0.0044	0.0057	0.3046	0.6354	0.5602
Kcnq1	0.0034	0.0028	0.0013	0.0012	0.0008	0.0005	0.6593	0.1035	0.1499
Kcnq2	0.1926	0.1619	0.1599	0.0139	0.0126	0.0112	0.1245	0.0866	0.9086
Kcnq3	0.0111	0.0082	0.0076	0.0014	0.0017	0.0011	0.2157	0.0677	0.7372
Kcns1	0.0011	0.0011	0.0006	0.0003	0.0002	0.0001	0.9076	0.1635	0.0298
Ryr3	0.0207	0.0209	0.0180	0.0016	0.0017	0.0007	0.9265	0.1315	0.1290
Scn10a	0.0114	0.0118	0.0087	0.0026	0.0025	0.0010	0.9313	0.3197	0.2799
Scn11a	0.0007	0.0007	0.0006	0.0002	0.0001	0.0001	0.7959	0.5907	0.7158
Scn1a	0.1185	0.1128	0.1013	0.0108	0.0129	0.0084	0.7427	0.2252	0.4678
Scn1b	0.4505	0.3967	0.3220	0.0709	0.1124	0.0512	0.7025	0.1586	0.5549
Scn2a1	0.8483	0.7664	0.7392	0.0419	0.0546	0.0381	0.2663	0.0757	0.6889
Scn2b	0.6113	0.5373	0.5553	0.0250	0.0166	0.0273	0.0254	0.1590	0.5825
Scn3a	0.1998	0.2069	0.2228	0.0181	0.0243	0.0137	0.8239	0.3215	0.5762
Scn8a	0.0729	0.0608	0.0736	0.0052	0.0045	0.0092	0.0990	0.9512	0.2299
Scn9a	0.0141	0.0153	0.0163	0.0009	0.0011	0.0018	0.4098	0.3050	0.6360
Slc12a5	0.4274	0.3429	0.3209	0.0347	0.0290	0.0260	0.0820	0.0268	0.5806
Trpa1	0.0006	0.0005	0.0009	0.0001	0.0001	0.0001	0.1739	0.0643	0.0044
Trpc1	0.0412	0.0401	0.0396	0.0015	0.0022	0.0024	0.7048	0.6047	0.8756
Trpc3	0.0409	0.0386	0.0300	0.0051	0.0087	0.0035	0.8261	0.0948	0.3769
Trpc6	0.0058	0.0061	0.0058	0.0001	0.0004	0.0004	0.5527	0.9868	0.6085
Trpm1	0.0002	0.0005	0.0003	0.0001	0.0003	0.0001	0.3178	0.1152	0.5850
Trpm2	0.0173	0.0156	0.0148	0.0008	0.0008	0.0007	0.1469	0.0376	0.4636
Trpm6	0.0017	0.0020	0.0019	0.0003	0.0005	0.0003	0.5994	0.6439	0.8532
Trpm8	0.0018	0.0018	0.0016	0.0002	0.0002	0.0002	0.9714	0.5881	0.6049
Trpv1	0.0031	0.0033	0.0029	0.0002	0.0003	0.0002	0.7456	0.3985	0.3266
Trpv2	0.0426	0.0336	0.0345	0.0027	0.0028	0.0015	0.0382	0.0183	0.7590
Trpv3	0.0039	0.0045	0.0049	0.0004	0.0005	0.0005	0.3170	0.1465	0.6086
Trpv4	0.0031	0.0025	0.0011	0.0012	0.0010	0.0006	0.6999	0.1659	0.2792

NR: no restraint, *n* = 7; 1D: 1-day restraint, *n* = 8; 5D: 5-day restraint, *n* = 8

**Table 2:** Voltage-gated ion channel mRNA expression in female rats following restraint

Females	Mean			SEM			*p* value		
Gene	NR	1D	5D	NR	1D	5D	NR vs. 1D	NR vs. 5D	1D vs. 5D
Accn1	0.1751	0.1158	0.1168	0.0237	0.0103	0.0099	0.0292	0.0310	0.9410
Accn2	0.0617	0.0756	0.0823	0.0083	0.0059	0.0074	0.1745	0.0743	0.4873
Accn3	0.0005	0.0006	0.0006	0.0001	0.0002	0.0001	0.5300	0.5813	0.8412
Best1	0.0007	0.0008	0.0008	0.0002	0.0002	0.0001	0.7402	0.5476	0.8681
Cacna1a	0.0349	0.0211	0.0128	0.0084	0.0022	0.0013	0.1145	0.0229	0.0096
Cacna1b	0.0748	0.0432	0.0298	0.0165	0.0060	0.0032	0.0767	0.0127	0.0711
Cacna1c	0.0013	0.0022	0.0029	0.0005	0.0009	0.0008	0.3552	0.1042	0.5907
Cacna1d	0.0053	0.0076	0.0095	0.0011	0.0014	0.0009	0.1963	0.0077	0.2777
Cacna1g	0.1486	0.1914	0.1925	0.0132	0.0288	0.0221	0.1933	0.1052	0.9771
Cacna1i	0.2557	0.2178	0.2582	0.0385	0.0213	0.0502	0.4052	0.9690	0.4985
Cacnb1	0.0004	0.0006	0.0006	0.0002	0.0002	0.0002	0.4759	0.2995	0.7909
Cacnb2	0.2809	0.2685	0.2148	0.0271	0.0275	0.0324	0.7446	0.1302	0.2268
Cacnb3	0.0657	0.0919	0.1077	0.0315	0.0357	0.0272	0.5794	0.3128	0.7308
Cacng2	0.1404	0.1519	0.1496	0.0128	0.0175	0.0177	0.5957	0.6704	0.9299
Cacng4	0.0606	0.0906	0.1013	0.0186	0.0117	0.0084	0.1746	0.0540	0.4689
Clcn2	0.0110	0.0153	0.0212	0.0037	0.0032	0.0018	0.3803	0.0211	0.1268
Clcn3	0.1296	0.1584	0.1630	0.0143	0.0119	0.0067	0.1311	0.0426	0.7389
Clcn7	0.0317	0.0477	0.0534	0.0064	0.0073	0.0023	0.1117	0.0072	0.4982
Hcn1	0.0010	0.0017	0.0031	0.0004	0.0007	0.0005	0.3918	0.0084	0.1688
Hcn2	1.0199	0.7647	0.7077	0.1448	0.0602	0.0776	0.1072	0.0653	0.5709
Kcna1	0.1321	0.1141	0.1280	0.0305	0.0129	0.0184	0.5736	0.9057	0.5454
Kcna2	0.2605	0.3659	0.4218	0.0490	0.0407	0.0232	0.1217	0.0108	0.2860
Kcna5	0.0044	0.0069	0.0090	0.0011	0.0027	0.0017	0.3963	0.0378	0.5145
Kcna6	0.2098	0.2631	0.3011	0.0185	0.0228	0.0290	0.0842	0.0171	0.3201
Kcnab1	0.2194	0.2096	0.1976	0.0387	0.0192	0.0265	0.8135	0.6344	0.7201
Kcnab2	1.0260	0.6351	0.5864	0.2262	0.0673	0.0678	0.1001	0.0679	0.6186
Kcnab3	0.0955	0.0728	0.0682	0.0135	0.0101	0.0096	0.1819	0.1066	0.7421
Kcnb1	0.3392	0.2701	0.3246	0.0425	0.0265	0.0357	0.1706	0.7893	0.2409
Kcnb2	0.0336	0.0421	0.0521	0.0048	0.0035	0.0024	0.1605	0.0029	0.0336
Kcnc1	0.1273	0.1293	0.1159	0.0159	0.0130	0.0147	0.9193	0.5945	0.5044
Kcnc2	0.1012	0.1690	0.1802	0.0278	0.0355	0.0308	0.1453	0.0701	0.8152
Kcnd2	0.5412	0.4421	0.3501	0.0542	0.0266	0.0157	0.1052	0.0029	0.0100
Kcnd3	0.0107	0.0181	0.0228	0.0036	0.0042	0.0033	0.1867	0.0221	0.3953
Kcnh1	0.0315	0.0342	0.0511	0.0057	0.0049	0.0083	0.7283	0.0670	0.1194
Kcnh2	0.0074	0.0127	0.0157	0.0019	0.0028	0.0026	0.1394	0.0204	0.4467
Kcnh3	0.0001	0.0000	0.0001	0.0000	0.0000	0.0000	0.4259	0.3606	0.1762
Kcnh6	0.0025	0.0028	0.0031	0.0007	0.0005	0.0004	0.7256	0.4128	0.5988
Kcnh7	0.0016	0.0029	0.0040	0.0007	0.0009	0.0011	0.2745	0.0888	0.4573
Kcnj1	NA	NA	NA	NA	NA	NA	NA	NA	NA
Kcnj11	0.0011	0.0020	0.0031	0.0006	0.0008	0.0009	0.3593	0.0825	0.3694
Kcnj12	0.0459	0.0481	0.0574	0.0034	0.0043	0.0063	0.6878	0.1295	0.2472
Kcnj13	0.0099	0.0048	0.0049	0.0055	0.0031	0.0031	0.4044	0.4192	0.9729
Kcnj14	0.0250	0.0209	0.0269	0.0032	0.0009	0.0043	0.2056	0.7120	0.1895
Kcnj15	0.0001	0.0001	0.0002	0.0000	0.0001	0.0001	0.3565	0.2888	0.5972
Kcnj16	0.2982	0.2624	0.1824	0.0510	0.0215	0.0109	0.5045	0.0454	0.0078
Kcnj2	0.0067	0.0073	0.0077	0.0011	0.0010	0.0009	0.6919	0.4787	0.7864
Kcnj3	0.0558	0.0793	0.0825	0.0071	0.0100	0.0100	0.0708	0.0432	0.8232
Kcnj4	NA	NA	NA	NA	NA	NA	NA	NA	NA
Kcnj5	0.0011	0.0014	0.0017	0.0004	0.0004	0.0002	0.5693	0.2218	0.5382
Kcnj6	0.0127	0.0113	0.0147	0.0012	0.0009	0.0014	0.3485	0.2862	0.0701
Kcnj9	0.6001	0.4530	0.4145	0.1062	0.0535	0.0485	0.2133	0.1155	0.6030
Kcnk1	0.0808	0.1219	0.1527	0.0204	0.0149	0.0082	0.1119	0.0062	0.1102
Kcnma1	0.0398	0.0484	0.0567	0.0176	0.0137	0.0101	0.6955	0.3960	0.6328
Kcnmb4	0.5967	0.3840	0.3054	0.0765	0.0240	0.0318	0.0136	0.0023	0.0684
Kcnn1	0.0031	0.0044	0.0063	0.0005	0.0007	0.0004	0.1499	0.0001	0.0358
Kcnn2	0.0371	0.0496	0.0559	0.0033	0.0036	0.0043	0.0203	0.0042	0.2900
Kcnn3	0.0136	0.0219	0.0314	0.0048	0.0046	0.0030	0.2122	0.0075	0.1206
Kcnq1	0.0027	0.0021	0.0018	0.0006	0.0006	0.0007	0.4491	0.2832	0.7516
Kcnq2	0.0355	0.0526	0.0743	0.0151	0.0152	0.0071	0.4256	0.0282	0.2180
Kcnq3	0.0004	0.0008	0.0012	0.0002	0.0004	0.0004	0.3837	0.0898	0.4585
Kcns1	0.0007	0.0009	0.0014	0.0002	0.0002	0.0003	0.3570	0.0618	0.2056
Ryr3	0.0153	0.0167	0.0195	0.0014	0.0014	0.0009	0.4745	0.0198	0.1235
Scn10a	0.0010	0.0010	0.0014	0.0002	0.0002	0.0003	0.8935	0.2895	0.2872
Scn11a	0.0001	0.0002	0.0003	0.0001	0.0001	0.0001	0.4579	0.2851	0.6279
Scn1a	0.5777	0.4378	0.4425	0.1146	0.0445	0.0391	0.2478	0.2557	0.9371
Scn1b	2.5908	2.0979	1.9782	0.4956	0.2079	0.2676	0.3485	0.2717	0.7292
Scn2a1	0.0186	0.0462	0.0722	0.0041	0.0129	0.0094	0.0768	0.0003	0.1447
Scn2b	0.2662	0.3831	0.4718	0.0478	0.0422	0.0255	0.0783	0.0013	0.0938
Scn3a	0.0141	0.0216	0.0291	0.0045	0.0048	0.0057	0.2557	0.0522	0.3295
Scn8a	0.0852	0.1306	0.1654	0.0143	0.0123	0.0148	0.0315	0.0013	0.1057
Scn9a	0.0108	0.0095	0.0096	0.0020	0.0011	0.0019	0.5430	0.6701	0.9523
Slc12a5	0.0729	0.1907	0.2675	0.0039	0.0511	0.0287	0.0721	0.0001	0.2361
Trpa1	0.0028	0.0026	0.0023	0.0004	0.0005	0.0005	0.7403	0.4882	0.7460
Trpc1	0.0256	0.0317	0.0358	0.0030	0.0016	0.0020	0.0808	0.0100	0.1220
Trpc3	0.0693	0.0676	0.0661	0.0085	0.0085	0.0097	0.8880	0.8015	0.9069
Trpc6	0.0068	0.0073	0.0078	0.0007	0.0009	0.0015	0.6690	0.5567	0.7857
Trpm1	0.0009	0.0003	0.0002	0.0003	0.0001	0.0000	0.0664	0.0940	0.5983
Trpm2	0.0789	0.0534	0.0477	0.0154	0.0068	0.0066	0.1331	0.0696	0.5534
Trpm6	0.0051	0.0059	0.0053	0.0007	0.0007	0.0009	0.4047	0.8921	0.5836
Trpm8	0.0081	0.0060	0.0051	0.0019	0.0009	0.0005	0.3147	0.1309	0.4378
Trpv1	0.0058	0.0064	0.0050	0.0009	0.0010	0.0005	0.6634	0.3947	0.2055
Trpv2	0.0114	0.0142	0.0128	0.0018	0.0026	0.0014	0.3978	0.5365	0.6633
Trpv3	NA	NA	NA	NA	NA	NA	NA	NA	NA
Trpv4	0.0082	0.0037	0.0032	0.0024	0.0012	0.0008	0.1021	0.0580	0.7375

NR: no restraint, *n* = 8; 1D: 1-day restraint, *n* = 8; 5D: 5-day restraint, *n* = 8; NA: no amplification

Compared to non-restrained controls, female rats that were restrained for 5 days displayed increased mRNA of Kcnb2, which encodes the second member of the voltage-gated potassium channel subunit subfamily B (Fig. 6D, *F*(2,20) = 6.666, *p* = 0.0057; post-hoc *p* = 0.0029). Expression of Kcnk1 mRNA was increased 24 h following a 5th restraint compared to non-restrained females (Fig. 6E, *F*(2,20) = 5.435, *p* = 0.01; post-hoc *p* = 0.0072). Female rats also displayed increased expression of the calcium-activated potassium channels Kcnn1 (Fig. 6F, *F*(2,20) = 8.786, *p* = 0.0017; post-hoc *p* = 0.0009) and Kcnn2 (Fig. 6G, *F*(2,20) = 6.448, *p* = 0.0069; post-hoc *p* = 0.0043) 24 h following a 5th restraint compared to non-restrained females. Expression of a different calcium-activated potassium channel, Kcnmb4, was reduced 24 h following 1 and 5 days of restrained compared to non-restrained females [Fig. 6H, *F*(2,20) = 10.17, *p* = 0.0008; post-hoc *p* = 0.0084 (1 day), *p* = 0.0006 (5 days)]. Expression of Kcnd2, which encodes the second member of the voltage-gated potassium channel subfamily D, was decreased 24 h following a 5th restraint compared to non-restrained females (Fig. 6I, *F*(2,20) = 7.782, *p* = 0.003; post-hoc *p* = 0.001). Compared to non-restrained females, mRNA expression of voltage-gated sodium channel subunit-encoding transcripts Scn2a1 (Fig. 6J, *F*(2,20) = 6.689, *p* = 0.0063; post-hoc *p* = 0.0032), Scn2b (Fig. 6K, *F*(2,20) = 7.187, *p* = 0.0042; post-hoc *p* = 0.0021), and Scn8a (Fig. 6L, *F*(2,20) = 8.776, *p* = 0.0018; post-hoc *p* = 0.0009) were increased in female rats 24 h following a 5th restraint. Expression of Slc12a5, which encodes a potassium–chloride co-transporter, was increased in females 24 h following 5 days of restraint compared to non-restrained controls (Fig. 6M, *F*(2,20) = 5.951, *p* = 0.01; post-hoc *p* = 0.0056). These findings demonstrate that restraint alters the expression of mRNA transcripts that encode voltage-gated ion channels and related proteins in the pPVT. Furthermore, repeated restraint alters the expression of different voltage-gated ion channels in males and females. Of the channels in which significant differences were observed, the expression of most were similarly impacted by either 1 or 5 days of restraint. Kcnk1 was the only channel that displayed altered expression following 1, but not 5, days of restraint.

## Discussion

Here we demonstrate that pPVT neurons in males and females display different synaptic, passive membrane, and active membrane properties in response to restraint stress. Identification of these differences in PVT neuron function represents important progress in our understanding of sex differences in the stress response as the pPVT is necessary for habituation to repeated restraint stress [7, 33]. In general, our findings demonstrate that electrophysiological properties of pPVT neurons are altered by restraint in males, but females only display moderate changes in response to restraint. Changes in the mRNA of specific voltage-gated ion channel transcripts within the pPVT are consistent with electrophysiological properties of pPVT neurons that are altered by restraint and/or different between males and females. Together, these results are the first to identify sex differences in pPVT neuron function in the context of stress.

### Males and females display differences in sEPSCs in the pPVT at baseline and following restraint

We examined the effects of restraint on electrophysiological properties of pPVT neurons in males and females. Electrophysiological properties were assessed 24 h following 1 or 5 days of restraint rather than immediately following restraint as we were primarily interested in investigating stable changes induced by stress. Examination of pPVT neurons proximal to restraint is likely to identify properties of these cells that are impacted by the immediate stress or rapid recovery from the stress. While the short-term impact of stress is important to study, we chose to examine those properties of pPVT cells that are likely to influence the response to stress the next day as habituation is influenced by prior experience and predictability of stress experiences [1]. We found that sEPSC amplitude was lower in non-restrained males compared to non-restrained females. Restraint, either 1 or 5 days, increased sEPSC amplitude in males to be similar to that of non-restrained females and had no effect on sEPSC amplitude in females. Increased EPSC amplitude can be primarily attributed to increased post-synaptic glutamate receptors. However, other factors may also contribute to increases in EPSC amplitude including increases in other neurotransmitter receptors with high cation conductance or increased pre-synaptic release of glutamate. Although we cannot rule out these possibilities, the observed decay times suggest that sEPSCs in the pPVT are consistent with those of AMPA receptors [40]. Therefore, we hypothesize that restraint increases AMPA receptor expression or trafficking to the synapse in male, but not female, pPVT neurons.

The sEPSC decay times of 4–10 ms that we observed in pPVT neurons were consistent with those of AMPA receptors [40], whereas those of NMDA, kainate, and metabotropic receptors are significantly longer and in the 100 ms range [41–46]. We observed that sEPSC decay time is increased in non-restrained females compared to non-restrained males. sEPSC decay time is increased by 1 and 5 days of restraint in males, but decreased by 1 and 5 days of restraint in females. Increased EPSC decay time increases excitatory post-synaptic potential amplitude and increases the probability of action potential firing [47]. Therefore, compared to baseline conditions, restraint is predicted to increase action potential firing in pPVT neurons of males, but reduce action potential firing in pPVT neurons of females. Longer sEPSC decay times may be attributed to asynchronous glutamate release in pre-synaptic axon terminals, increased glutamate release, or slower glutamate reuptake and clearance from the synaptic cleft [48, 49]. Thus, glutamate transporter expression or function may be decreased by restraint in males, but increased by restraint in females. There were no effects of sex or restraint on sEPSC frequency. Together, these findings demonstrate that both sEPSC amplitude and decay time are increased by restraint in males, but not females. Both of these electrophysiological properties correlate with increased action potential firing probability [47]. This is consistent with our recent finding that expression of the neuronal activity markers c-Fos and activity-regulated cytoskeleton-associated protein (Arc) is increased in the pPVT of male rats following 1 or 5 days of restraint [17]. Thus, increased sEPSC amplitude and decay time represent two important electrophysiological properties that are consistent with increases in pPVT action potential firing caused by restraint in males. Because pPVT activity is necessary for habituation [7, 17], these electrophysiological properties may contribute to habituation in male rats.

Increased EPSC amplitude and decay time might be indicative of glutamate-mediated reformatting of pPVT neurons. We recently demonstrated that in the pPVT, Arc, which is increased by glutamatergic input [50–53], is necessary for habituation in male rats. We showed that Arc promotes habituation by increasing spine density [17]. Arc also regulates long-term depression [51] and synaptic scaling [54], a form of homeostatic plasticity that prevents increases in overall action potential firing rates. Therefore, Arc reformats neurons by increasing the strength of some synapses but weakening other synapses. We hypothesize that this reformatting of pPVT neurons is critical for habituation. Because sEPSC amplitude and decay time are similar in males and females following 5 restraints, these properties in isolation are probably not critical mechanisms underlying habituation. However, increases in sEPSC amplitude and decay time compared to non-restrained controls, which only occurs in males, may promote habituation as it may be an important factor underlying the reformatting of pPVT neurons. In female rats, sEPSC amplitude is unchanged by restraint and sEPSC decay time is reduced by restraint. These findings are predicted to have no effect on pPVT firing or reduce pPVT firing, respectively, and may at least partially explain why habituation is impaired in female rats.

### Males and females display differences in passive membrane properties in the pPVT at baseline and following restraint

Resting membrane potential was more hyperpolarized in female pPVT neurons compared to males. Although the difference in mean resting membrane potential was modest, even subtle changes in this basic electrophysiological property could affect action potential firing. Resting membrane potential is regulated by differences in extracellular and intracellular ion concentrations and the permeability of those ions [55, 56]. Therefore, more hyperpolarized pPVT neurons in females may be due to changes in ion transporters or ion leak channels. Resting membrane potential in pPVT neurons is complex and regulated by a wide variety of conductances including those mediated by inward rectifying potassium channels and TWIK-related acid sensitive potassium channels [57]. Thus, increases in the expression and/or membrane trafficking of these ion channels and others may underlie the more hyperpolarized resting membrane potential observed in the pPVT neurons of females compared to males. Input resistance was increased by 1 and 5 days of restraint in pPVT neurons of male, but not female, rats. Increased input resistance is primarily attributed to reduced potassium leakage in response to depolarizing current. Restraint may reduce the expression, function, and/or membrane trafficking of potassium leak channels in pPVT neurons of male, but not female, rats. Reduced potassium leakage in response to membrane depolarization is predicted to facilitate EPSC integration and thus increase the likelihood of action potential firing. Together, these findings suggest that compared to females, the passive membrane properties of pPVT neurons of male rats make them more likely to fire action potentials in response to excitatory inputs. Therefore, in addition to restraint-induced increases in sEPSC amplitude and decay time, a more depolarized resting membrane potential and restraint-induced increases in input resistance may further contribute to the observation that pPVT activity is increased by restraint in male rats [17]. Resting membrane potential is more hyperpolarized in females compared to males and restraint has no effect on input resistance in females. These findings are predicted to render pPVT activity less responsive to restraint and may contribute to habituation impairments in females.

### Males and females display differences in active membrane properties in the pPVT at baseline and following restraint

Males displayed higher percentages of single spike and burst-firing pPVT neurons than females, whereas females displayed a higher percentage of sustained-firing neurons. These findings were consistent regardless of restraint group. Sustained-firing neurons have a short refractory period, allowing them to fire continuously in response to prolonged experimental depolarization. Because sustained-firing neurons fire action potentials with a higher frequency in response to continuous depolarization, a higher percentage of sustained firing pPVT neurons in females might suggest increased pPVT output in females compared to males. However, this would only be true if the pPVT is continuously depolarized in vivo. Excitatory inputs that depolarize pPVT neurons past the action potential threshold may occur less frequently in vivo than the refractory period required for action potential firing in burst-firing pPVT neurons. If this was the case, action potential frequency in pPVT neurons might be similar in males and females, because the limiting factor in firing an action potential would be frequency and amplitude of EPSCs. The in vivo refractory period of burst-firing pPVT neurons and in vivo EPSC frequency are unknown to the best of our knowledge. Therefore, we cannot accurately predict the effect of neuron firing type percentages on overall pPVT action potential firing frequency in vivo.

Within each sex, restraint had little effect on the percentage of pPVT neurons displaying properties of each firing type. Compared to the other female groups, the 5-day restraint females exhibited an increased percentage of sustained-firing neurons and a decrease in bursting neurons. However, we should note that chi-square analysis was only significant when all groups were included in the analysis. We did not observe differences between any two groups within or between sexes. Firing patterns of pPVT neurons change from day to night phases. pPVT neurons during the light phase are more hyperpolarized and primarily single-spiking, but during the dark phase they are more depolarized and tend to be bursting or sustained-firing. These changes during the dark phase are regulated by reduced potassium currents and increased T-type calcium channel currents [57, 58]. We should note that these recordings were taken from anterior PVT neurons, whereas our recordings were taken from posterior PVT neurons and our recordings were conducted during the animal’s light phase. This is notable, because anterior and posterior subdivisions of the pPVT have different functions and anatomical connectivity [20, 29–31]. Furthermore, the effects of sex on circadian influence of pPVT neuron firing types is unknown to the best of our knowledge.

Following 1, but not 5, days of restraint, the threshold for firing an action potential in pPVT neurons of males was modestly depolarized compared to non-restrained controls. We also observed an overall sex effect indicating that action potential firing threshold was more depolarized in male pPVT neurons compared to females. These findings suggest that pPVT neurons of 1-day restraint males may require more excitatory input to fire an action potential compared to females. However, the resting membrane potential of male pPVT neurons is depolarized compared to females, so similar excitatory inputs may induce similar action potential firing in males and females in vivo. This is because the relative difference between resting membrane potential and the membrane potential required for firing action potentials (action potential firing threshold) is similar in males and females (~ 24 mV for each sex regardless of restraint group). Action potential half-width in pPVT neurons was increased in males restrained for 1, but not 5, days compared to non-restrained males. This indicates that 1 day of restraint increased the duration of action potentials in males, which may increase voltage-sensitive calcium transients in axon terminals and promote neurotransmitter release from pPVT neurons. Afterhyperpolarization potentials (AHPs) were greater in females compared to males, regardless of restraint group. This suggests that voltage-gated potassium channels may remain open longer in female pPVT neurons and the action potential refractory period may be longer compared to that of males. Although female pPVT neurons are more likely to display sustained firing patterns, greater AHPs may reduce action potential firing frequency in female pPVT neurons compared to male pPVT neurons with the same firing pattern. Together, these findings suggest that active membrane properties of pPVT neurons are different in males and females at baseline and in response to restraint stress.

### Restraint stress has different effects on the expression of voltage-gated ion channel mRNA transcripts in the pPVT of males and females

Similar to the timeframes of our studies on the electrophysiological properties of male and female pPVT neurons, we examined the mRNA of voltage-gated ion channel transcripts 24 h following 1 and 5 days of restraint compared to non-restrained controls. This allowed us to investigate stable changes in gene expression that temporally correlate with changes in pPVT neuron electrophysiology. All data were presented relative to mean housekeeping gene expression of non-restrained controls, so that relative levels of each transcript, and, therefore, their influence on neuron function, could be inferred qualitatively. Male rats displayed reduced expression of Kcnj6 transcripts 24 h following a 5th daily restraint. Kcnj6 encodes the G-protein activated inward rectifier potassium channel 2. Inwardly rectifying potassium currents are important regulators of the resting membrane potential of pPVT neurons [57]. Therefore, reduced expression of Kcnj6 may contribute to depolarization of resting membrane potential in males following 5 days of restraint. Although resting membrane potential of pPVT neurons is not changed by restraint in males, reduced Kcnj6 expression may be an important factor that compensates for other factors that would otherwise drive hyperpolarization of resting membrane potential following restraint. Wild-type Kcnj6 inhibits dopaminergic tone [59–61]. A Kcnj6 variant has been linked to alcohol dependence in individuals exposed to psychosocial stress early in life [62]. Single nucleotide polymorphisms (SNPs) in the Kcnj6 gene are risk factors for developing attention–deficit/hyperactivity disorder (ADHD) [63]. Addiction [64, 65] and ADHD [66] are both heavily influenced by the rewarding and reinforcing effects of dopaminergic neurotransmission. The PVT receives dopaminergic inputs [67] and is an important regulator of reward [32, 68, 69] and attention [17]. Therefore, reduced Kcnj6 expression could contribute to addiction and/or attention deficits in stressed animals.

Male rats displayed increased expression of Kcnh3 24 h following a 5th restraint. Kcnh3 encodes Kv12.2, a voltage-gated potassium channel subunit. Kv12.2 deletions reduce action potential firing threshold [70]. Thus, increased expression of Kv12.2 following restraint may contribute to the more depolarized action potential firing threshold observed in the PVT of restrained male rats. Kcnh3 knockout mice display improved memory and PFC-mediated attention [71]. Because restraint [33] and the pPVT [17] regulate PFC-mediated cognitive flexibility and attention, restraint-induced increases in Kcnh3 in the pPVT may impair cognitive function. Male rats displayed reduced expression of Kcnk1 mRNA, which encodes the two-pore domain potassium channel TWIK-1. Little is known about the function of TWIK-1 in the brain [72]. However, reduced Kcnk1 expression in 1-day restraint males may contribute to increased action potential half-width in 1-day restraint males as reduced Kcnk1 expression may impair potassium efflux. Together, these findings suggest that changes in the mRNA of certain voltage-gated ion channels in the pPVT of male rats may contribute to restraint-induced changes in the electrophysiological properties observed in the pPVT of male rats.

Although restraint did not affect active membrane properties in female pPVT neurons, mRNA transcripts encoding 10 different voltage-gated ion channels were altered by 5 days of restraint in the female pPVT. Some of these channels regulate similar functions, but restraint has opposite effects on their expression. Therefore, changes in the expression of these channels may be countered by compensatory changes in the expression of other transcripts encoding similar functions. For example, 24 h following a 5th daily restraint, female rats displayed increased mRNA expression of Kcnb2 transcripts, but decreased expression of Kcnd2. Kcnb2 and Kcnd2 encode the voltage-gated potassium channels Kv2.2 and Kv4.2, respectively. These channels regulate delayed rectifier currents during the action potential [73]. In addition, the expression of Kcnn1 and Kcnn2 transcripts, which encode the calcium-activated potassium channel subfamily N members KCa2.1 and KCa2.2 [74], respectively, were both increased 24 h following a 5th restraint in the female pPVT. These channels augment the afterhyperpolarization phase of the action potential and thereby have inhibitory effects in the neurons they are expressed in [75]. Kcnn2 overexpression in the amygdala reduces anxiety-like behavior, presumably by reducing amygdala output [76]. Restraint-induced increases in Kcnn2 might impair habituation in females by countering restraint-induced effects in the pPVT that would otherwise increase its excitability. The expression of Kcnmb4, which encodes the calcium-activated potassium channel subfamily M beta subunit 4, was decreased 24 h following 1 and 5 days of restraint in female pPVT neurons. It is possible that the opposing expression patterns of these calcium-activated potassium channels counter one another to prevent restraint-induced changes in afterhyperpolarization potential.

Compared to non-restrained controls, the expression of Scn2a1, Scn2b, and Scn8a were all increased in the PVT of females 24 h following 5 days of restraint. Scn2a1, Scn2b, and Scn8a encode the voltage-gated sodium channel subunits Nav1.2, Navβ2, and Nav1.6, respectively [77]. Both Nav1.1 and Nav1.6 contain voltage-sensing and pore-forming domains of the voltage-gated sodium channel [78]. Navβ2 is an auxiliary subunit for voltage-gated sodium channels involved in the trafficking of voltage-sensitive subunits to the plasma membrane and stabilizing it there [78–80]. Although 3 different transcripts that are predicted to increase voltage-dependent sodium channel currents were increased in the pPVT of females restrained for 5 days compared to non-restrained females, no active membrane properties were affected by restraint in females. This may be because voltage-gated sodium channel translation was impaired, trafficking of the channels to the plasma membrane was impaired, or compensatory changes in the functional expression of other channels that regulate active membrane properties negated voltage-gated sodium channel function. Compared to non-restrained controls, the expression of Kcnk1 was increased in females 24 h following a 5th daily restraint. The expression of Slc12a5, which encodes the potassium–chloride cotransporter KCC2, was increased in the pPVT of females following 5 restraints compared to non-restrained controls. This channel is the major extruder of intracellular chloride in mature neurons, allowing for chloride influx during GABAergic neurotransmission [81]. Therefore, increased expression of KCC2 could enhance GABAergic chloride currents that inhibit PVT activity in vivo. Together, these findings indicate that although restraint did not affect active membrane properties of PVT neurons in females, the expression of 10 voltage-gated ion channels were altered by restraint. Changes in some of these transcripts may contribute to the restraint-induced changes in firing patterns observed in females. Effects of altered mRNA expression may be negated by impaired translation, impaired protein regulation, or countered by compensatory changes in the expression or function of other voltage-gated ion channels as has been reported in rodent models of epilepsy [82]. Further studies are needed to fully understand how these stress-induced changes in expression of voltage gated ion channels may regulate functional consequences of pPVT activity in male and female animals.

## Conclusions

These findings are the first to characterize the effects of sex and stress on electrophysiological properties and voltage-gated ion channel expression in pPVT neurons. We found that restraint altered EPSCs, input resistance, and active membrane properties that are predicted to increase pPVT activity in males, but restraint did not have these effects in females. In pPVT neurons of males, restraint-induced changes in the expression of certain voltage-gated ion channels may contribute to some of the changes in active membrane properties that were altered by restraint. Restraint modulated the expression of 10 different voltage-gated ion channels in pPVT neurons of females, but there were few effects of stress on electrophysiological properties. Together, these findings suggest that restraint increases pPVT activity in males, but only modestly alters pPVT activity in females. These findings identify mechanisms through which males may habituate to 5 days of repeated restraint but females do not. Sex differences in electrophysiological properties of pPVT neurons, under baseline conditions and following restraint, may underlie impaired habituation in females. The increased number of voltage-gated ion channels that are altered by restraint in females might reflect compensatory changes in channel expression that prevent altered electrophysiological function, which might have been caused by restraint-induced changes in the expression of a few key voltage-gated ion channels. Restraint-induced changes in ion channel expression might also represent flux in membrane properties that could become apparent once females begin to habituate, which is likely to occur around day 9 or 10. These changes may also be due to differences in estrous cyclicity, which we did not investigate here. Future studies will determine the role of estradiol on voltage-gated ion channel expression using ovariectomized females and estradiol replacement. Because the pPVT is as important for facilitation to novel stress as it is for habituation, whether the changes observed here contribute to facilitation is not known. For a habituated animal to facilitate, the reduction in excitation in habituated animals would need to be overcome, through strong excitatory inputs that are initiated by the novel facilitating stressor. Alternatively, it is possible that the PVT cells in which activity is important for habituation are different than those whose activity is important for facilitation. These findings are an important step in developing a comprehensive understanding in the genes and electrophysiological processes that underlie habituation in male rats and the mechanisms contributing to impaired habituation in females.

## Perspectives and insights

Together, our findings suggest that restraint causes different effects on the electrophysiological properties of pPVT neurons in males compared to females. Restraint-induced increases in the synaptic strength of inputs to the pPVT may allow male rats to habituate more effectively. Given the importance of the pPVT in regulating habituation, our findings may, at least in part, offer an explanation as to why male rats habituate more effectively compared to females. Future directions will investigate the anatomical inputs that drive electrophysiological changes in male rats. Furthermore, we plan to investigate whether these electrophysiological properties in pPVT neurons are also important for facilitation of the stress response.

## Data Availability

Please contact authors for data requests.

## Abbreviations

aCSF: Artificial cerebrospinal fluid
ACTH: Adrenocorticotropic hormone
ADHD: Attention–deficit/hyperactivity disorder
AHP: Afterhyperpolarization potentials
BNST: Bed nucleus of the stria terminalis
HPA: Hypothalamic pituitary adrenal
pPVT: Posterior division of the paraventricular thalamic nucleus
mPFC: Medial prefrontal cortex
PCR: Polymerase chain reaction
PTSD: Post-traumatic stress disorder
PVN: Paraventricular nucleus of the hypothalamus
sEPSC: Spontaneous excitatory post-synaptic current
